# Advances in polymer based Friedlander quinoline synthesis

**DOI:** 10.3906/kim-2106-5

**Published:** 2021-10-19

**Authors:** Rajendra PATIL, Jagdish CHAVAN, Shivnath PATEL, Anil BELDAR

**Affiliations:** 1 Department of Chemistry, P.S.G.V.P. M’s SIP Arts, GBP Science and STKVS Commerce College, Shahada, Nandurbar India

**Keywords:** Friedlander reaction, polymers, catalysts

## Abstract

Nitrogen containing heterocyclic compounds has acquired their remarkable and distinct place in the wide area of organic synthesis due to the broad range of applications. Among them, quinoline motifs have attracted researchers in the synthetic chemistry because of its presence in the large number of pharmacologically active compounds. Different methods for synthesis of quinoline derivatives are reported, among them the Friedlander synthesis have provided comparatively more efficient approach. Many of the reported conventional Friedlander methodologies have some problems such as difficult product isolation procedures, poor yields and use of expensive catalysts, etc. Recently, polymer or solid supported synthetic approaches have attracted the attention of researchers because of their easy execution, greater selectivity, increased product yields, simple work-up procedures, recoverability and reusability of the catalysts. In consideration with the advantages of polymer supported synthetic strategies, the proposed review covers the role of polymers in the Friedlander synthesis; which may use polymers of organic, inorganic or hybrid in nature and of nanolevel as well.

## 1. Introduction

**Figure 1:**
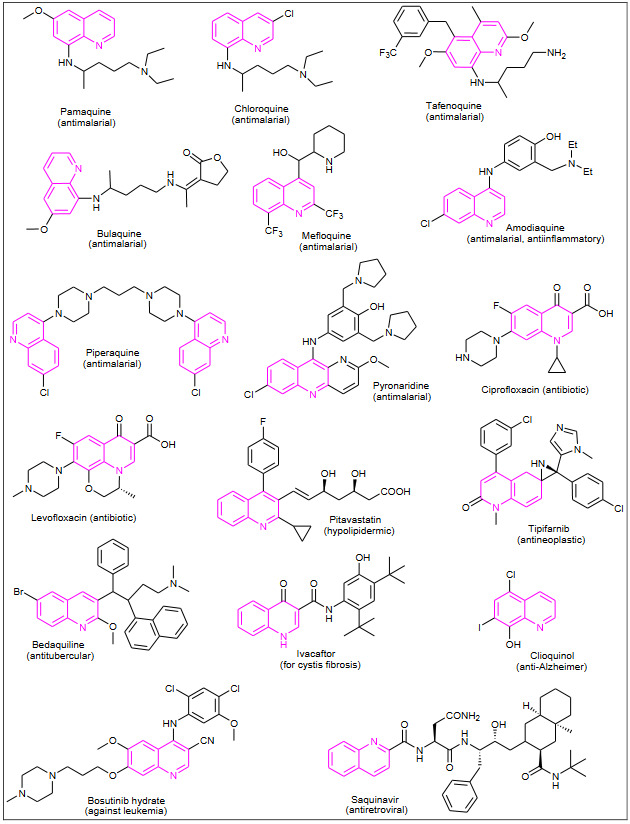
Quinoline pharmacophore containing drugs.

Quinoline scaffolds occur in large number of pharmacologically active compounds obtained from natural sources as well as prepared synthetically. The compounds containing quinoline moiety like pamaquine, chloroquine, tafenoquine, bulaquine, mefloquine, piperaquine, pyronaridine are the potent antimalarial agents and amodiaquine as an antimalarial and antiinflammatory agent. The quinoline derivatives ciprofloxacin and levofloxacin marketed as broad-spectrum antibiotics [1]. Quinoline derivatives has been reported to possess diverse variety of medicinal activities such as anticancer [2,3], antibacterial [4], DNA binding [5], antiproliferative [6], antitumor [7, 8], antitubercular [9], anticonvulsant, antihypertensive [10], antiinflammatory, cardiovascular [11], antimalarial, antiprotozoal [12], antifungal [13], antioxidant [14], anti-HIV agents [15] and for treatment of central nervous system diseases [16]. The varieties of drugs containing quinoline pharmacophore are shown in Figure 1.

### 1.1. Quinoline synthesis

#### 1.1.1.Aniline based quinoline synthesis (classical approaches)

**Scheme 1:**
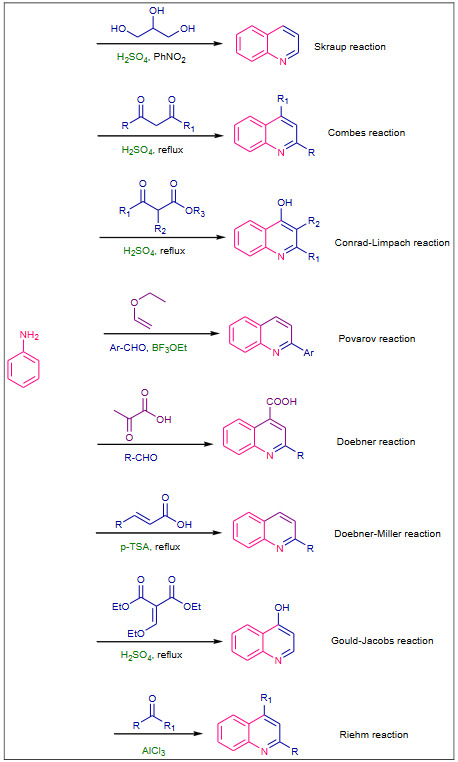
Aniline based classical routes for the synthesis of quinolines.

The first synthesis of quinoline was attempted using aniline as crucial reagent by Skraup (1880); align to this classical synthetic route several methods are reported as the advancement for synthesis of quinoline moieties. The synthetic methodologies which used aniline include Skraup reaction [17], Combes reaction [18], Conrad–Limpach reaction [19], Povarov reaction [20], Doebner reaction [21], Doebner–Miller reaction [22], Gould–Jacobs reaction [23] and Riehm reaction [24] (Scheme 1).

#### 1.1.2 Modification to the aniline based quinoline synthesis

**Scheme 2:**
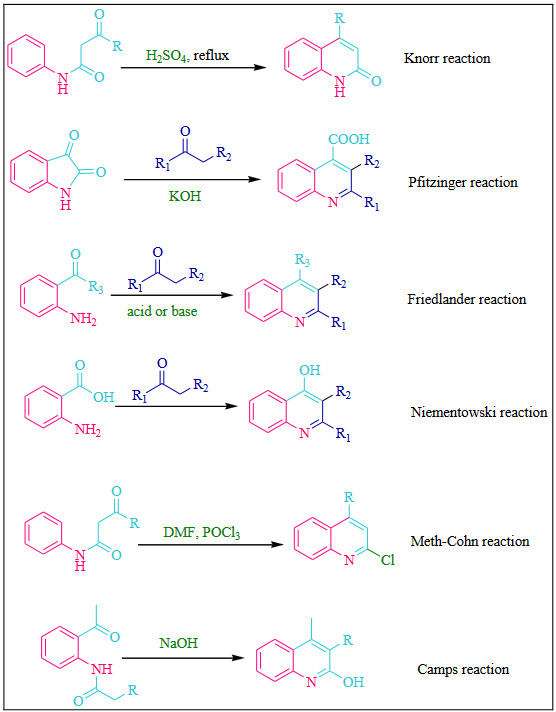
Substituted aniline based synthesis of quinolines [35].

Several synthetic routes were reported which involved modification to aniline based protocols. The substituted anilines or some typical substituted reactants are used for synthesis of quinoline motifs which includes Knorr reaction [25], Pfitzinger reaction [26], Friedlander reaction [27], Niementowski reaction [28], Meth–Cohn reaction [29] and Camps reaction [30] (Scheme 2).

### 1.2. Friedlander quinoline synthesis

**Scheme 3:**
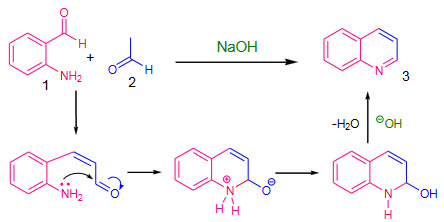
The classical Friedlander reaction with mechanism.

The Friedlander synthesis is one of the simplest methods among the several reported methods for the synthesis of quinoline (**3**) and its derivatives. The classical Friedlander reaction reported by Paul Friedlander in 1882, used 2-aminobenzaldehyde (**1**) with acetaldehyde (**2**) in the presence of sodium hydroxide [31] (Scheme 3). Generally, Friedlander reaction is carried out by refluxing aqueous or alcoholic reaction mixture in the presence of a base or an acid or by heating reaction mixture at higher temperature without catalyst [32]. Lewis acids were also used commonly and found efficient [33, 34]. 

### 1.3. Scope

The available literature reveals that some research groups have reported polymer supported solid phase synthesis [36,37] as well as polymers as catalyst and catalyst support [38] for synthesis of bioactive heterocyclic compounds. In this review, for the first-time efforts are made to compile all the literature regarding the role of polymers for the improvements in the Friedlander quinoline synthesis. The advancements to Friedlander synthesis include reagents supported on the polymers as well as polymers as catalyst and catalyst supports. Polymeric supports with variant composition, nature, size, properties were successfully investigated. Polymers of different origin are described under separate section such as organic polymers, inorganic polymers, hybrid polymers, biopolymers as biocatalysts. Also, the present article incorporated with diverse nanopolymers as catalysts. The review provides a bunch of recipes for the synthesis of heterocyclic moieties at one desk. Considering previous publications in the area of quinolines and their practical applications we trust the present review article will be of general interest to the readers.

## 2. Polymer supported reagent-based Friedlander quinoline synthesis

**Scheme 4:**
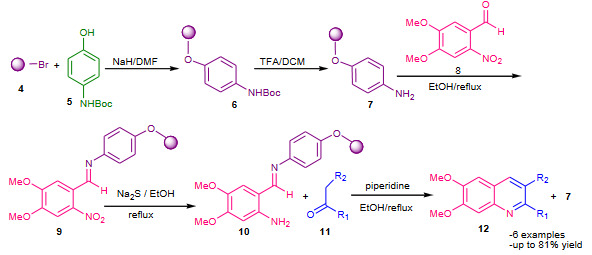
TentaGel resin supported modified Friedlander synthesis.

Patteux et al. (2003) [39] described the solid-phase synthesis of quinolines via a modified Friedlander reaction between the resin-bound imine (**10**) and ketones (**11**). The Boc-protected aminophenol (**5**) was treated with commercial TentaGel-Br resin (**4**) to afford resin** 6**, which on cleavage of Boc with TFA in DCM furnished the resin **7**. The supported imine **10** was prepared in two steps from resin **7** by treatment with 3,4-dimethoxy-6-nitrobenzaldehyde (**8**) in refluxing ethanol, affording resin-bound o-nitroimine (**9**) and subsequently on reduction of the nitro group in the presence of sodium sulfide in refluxing ethanol to obtain the desired resin-bond “masked” *o*-benzaldeimine (**10**). The resin bound imine (**10**) then treated with different ketones in the presence of piperidine under refluxing ethanol which underwent cyclative cleavage to yield substituted quinolines (**12**) (Scheme 4). Average to good yields of corresponding quinolines was obtained.

**Scheme 5:**
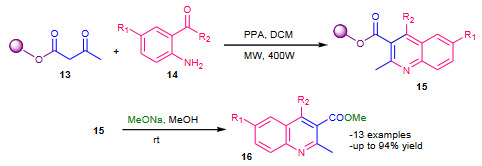
PEG supported synthesis of quinolines.

Zhang et al. (2011) described microwave assisted synthesis of polysubstituted quinolines using polyethylene glycol (PEG 4000) bound acetoacetate (**13**) with 2-aminoarylketones (**14**) in the presence of polyphosphoric acid (PPA) as a catalyst [40]. The PEG bound quinoline-3-carboxylates (**15**) were obtained from the reaction of **13** with **14**, which subsequently underwent cleavage from the PEG in the presence of MeONa in MeOH to afford polysubstituted quinolines **16** (Scheme 5). Excellent yields of products were reported. 

**Scheme 6:**
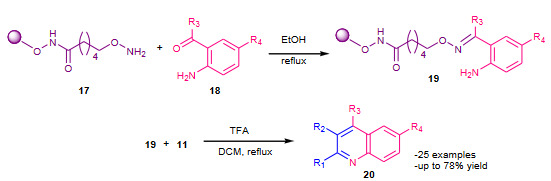
Alkoxyamine linker supported synthesis of quinolines.

Yamaguchi et al. (2015) reported solid phase Friedlander synthesis using an alkoxyamine linker [41]. The 2-aminobenzoyl compounds **18** were condensed with an alkoxyamine linker **17** on the solid support, affording resin-bound oximes **19**. The solid supported oximes **19** were then treated with ketones **11** in the presence of TFA and 1,2-dichloroethane (DCE) under refluxing conditions to afford quinolines **20** by a Friedlander-type reaction with cyclative cleavage of solid support (Scheme 6). 

## 3. Polymers based catalyst and as catalyst supports for Friedlander quinoline synthesis

### 3.1 Organic polymers

#### 3.1.1 Polymers and copolymers as catalyst and catalyst supports

**Figure 2:**
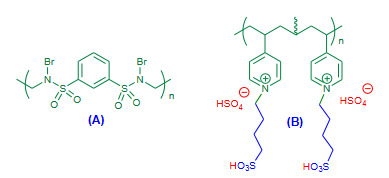
(A) PBBS reagent; (B) P(4VPBSA)-HSO4 catalyst.

Ghorbani-Vaghei and Akbari-Dadamahaleh (2009) achieved Friedlander synthesis under aqueous and solvent free conditions using poly(*N*-bromo-*N*-ethylbenzene-1,3-disulfonamide) [PBBS] (Figure 2A, Table 1) and *N*,*N*,*N*’,*N*”-tetrabromobenzene-1,3-disulfonamide [TBBDA] as efficient catalysts [42]. Both the aqueous and solvent free conditions affording excellent yields of products but, the aqueous protocol occurred in shorter time in comparison with to solvent free. 

**Table 1:** Organo-polymers based catalysts for optimized Friedlander reaction.

Types oforgano-polymer	Polymer supported catalysts	Amount of catalyst(ag, bmol %, c wt %)	Solvent	Energy source	Temp.(rt = room temp.)	Time(d h, e min, f s)	Yield(%)
Polymers and copolymers	PBBS	0.25a	Water	Heat	Reflux	6d	91
			-		100 °C	4d	84
	P(4VPBSA)-HSO4	10b	-		110 °C	45e	88
	Dendritic hydrocarbon and fluorocarbon chains	2b	-		80 °C	3d	91/93
	Poly(AMPS-co-AA)	0.06a	-		110 °C	10e	84
Polymericcarbon	Pd/C	5c/0.011a	Dioxane		100 °C	20d	87
	FeCl2·2H2O-RiHA	0.3a	-		90 °C	35e	86
	RHA-SO3H	0.08a	-		80 °C	30e	92
Resins	Dowex 2	0.5cc	Ethanol		Reflux	4d	69
	Amberlite IRA 400	0.5cc			Reflux	4d	89
	Amberlyst-15	10c	Ethanol		Reflux	2.5d	89
		0.1a	-		80 °C	3d	78
	Dowex-50W	0.1a	-		100 °C	30e	80
PEG	PEG-SO3H	0.45a	DCM		Reflux	40e	96
		3b	-	MW	130 °C	12e	81
		0.2a	Water	Heat	60 °C	15e	92
	NbCl5.PEG	0.1b	-		110 °C	15e	92
Cyclodextrin	β-CD	0.567a	Water		75 °C	6d	92

Kiasat et al. (2013) employed poly(4-vinylpyridiniumbutanesulfonic acid) hydrogensulfate [P(4VPBSA)-HSO_4_), Figure 2B, Table 1] as an efficient dual acidic catalyst for the one-pot synthesis of quinolines under solvent free conditions via Friedlander reaction [43]. The solvent free reaction was attempted in the presence of P(4VPBSA)-HSO_4 _as a catalyst. In spite of high temperature conditions, the method reported short reaction time and excellent yields.

**Figure 3:**
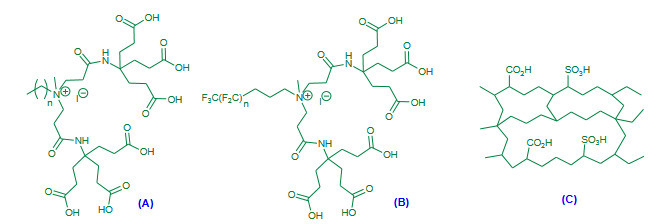
(A) Dendritic hydrocarbon chains as a catalyst; (B) Dendritic fluorocarbon as a catalyst; (C) Poly (AMPS-co-AA) catalyst.

Fang et al. (2013) investigated branched catalysts with hydrocarbon or fluorocarbon chains as a catalyst for Friedlander reaction under solvent free conditions [44]. The Friedlander components were heated neatly in the presence of dendritic hydrocarbon chains (Figure 3A, Table 1) or fluorocarbon chains (Figure 3B, Table 1) as a catalyst at 80 °C. After heterogeneous work up with ethyl acetate, good to excellent yields of corresponding substituted quinolines were obtained. Both the catalysts seem to be an equally efficient. The discussed methodology contributed new form of branched catalyst to the advancement in the Friedlander synthesis.

Maleki et al. (2015) investigated cross-linked polymer bound sulfonic acid [Poly (AMPS-co-AA), Figure 3C, Table 1] as a catalyst for synthesis of substituted quinolines via Friedlander reaction under solvent free conditions [45]. The catalyst Poly (AMPS-co-AA) was prepared from cross-linked *N*,*N*-methylene bis-acrylamide (MBA), 2-acrylamido-2-methylpropanesulfonic acid (AMPS) and acrylic acid (AA) by earlier reported method. Although the methodology involved high temperature conditions, it has been added new polymeric catalyst to the Friedlander catalyst library.

#### 3.1.2 Polymeric carbon as catalyst and catalyst supports

**Scheme 7:**
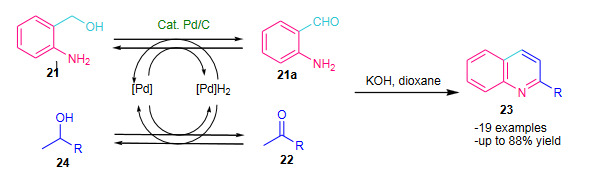
Pd/C catalysed modified Friedlander reaction.

Cho et al. (2005) reported Pd/C catalyzed modified Friedlander reaction using 2-aminobenzylalcohol (**21**) and ketones (**22**) in the presence of KOH using dioxane as solvent via oxidative cyclization (Scheme 7, Table 1) [46]. Although the method seems conventional with use of solvent, longer reaction times and less economic yields, but it has described the new modified Friedlander approach with the use of Pd/C as a catalyst.

Shirini et al. (2013) employed rice husk ash supported FeCl_2_·2H_2_O (FeCl_2_·2H_2_O-RiHA, Table 1) as a catalyst for the synthesis of multisubstituted quinolines by the Friedlander heteroannulation under solvent free conditions [47]. The method highlighted the entry of new carbon supported catalyst with excellent yields of products, short reaction times and reusability of catalyst for three cycles with negligible loss of activity.

The sulfonated rice husk ash (RHA-SO_3_H, Table 1) was reported as a catalyst for the Friedlander reaction reported by Shirini et al. (2015) under solvent free conditions [48]. The catalyst RHA-SO_3_H was prepared by the reaction between rice husk ash (RHA) and chlorosulfonic acid (ClSO_3_H). The recyclability of the catalyst for three consecutive cycles was mentioned. The method seems to be an efficient and provided high yields in short reaction times. 

#### 3.1.3 Resins as catalyst and catalyst supports

In 1955, Yamada and Chibata reported Dowex 2 and Amberlite IRA 400 (Table 1) resins as a catalyst for Friedlander quinoline synthesis [49]. This was a very first attempt of using ion exchange resins as a heterogeneous catalyst for the synthesis of substituted quinolines using Friedlander reaction. The Friedlander components were refluxed in ethanol in the presence of Amberlite IRA 400 or Dowex 2 resins as catalyst; good quantities of products were obtained. 

**Figure 4:**
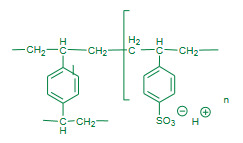
Amberlyst-15 catalyst.

Das et al. (2007) reported the Friedlander synthesis of substituted quinolines using Amberlyst-15 resin (Figure 4, Table 1) as a solid heterogeneous catalyst [50]. The Friedlander reactions were performed under refluxing ethanol in the presence of the Amberlyst-15 catalyst. The reusability of catalyst for three subsequent cycles was mentioned with slight declined activity. Although the method seems conventional; it has contributed a new form of catalyst to the advancements in the Friedlander reaction.

Hou et al. (2008) attempted ionic liquid mediated Friedlander quinoline synthesis using Amberlyst-15 (Figure 4, Table 1) resin as a catalyst. The method employed 1-*n*-butyl-3-methylimidazolium hexafluorophoaphate, [Bmin] [PF_6_] ionic liquid as a medium for the Friedlander reaction in the presence of catalyst Amberlyst-15 [51]. The methodology seems conventional although it has introduced new combination of catalyst and ionic liquid as a medium.

Wang et al. (2011) performed solvent free Friedlander annulation with an ion exchange resin, Dowex-50W as a catalyst[52]. The heterogeneous phase work up procedure affording good amount of product yields. Shorter reaction times with solvent free condition make the method efficient, but the high temperature requirement for the catalytic efficiency makes it more common.

#### 3.1.4. Polyethylene glycol (PEG) as catalyst and catalyst supports

Zhang et al. (2009) [53] reported Friedlander synthesis using poly(ethylene glycol) (PEG-4000)-supported sulfonic acid (PEG-SO_3_H, Table 1). The mixture of 2-aminoarylketone, α-methyleneketone and PEG-SO_3_H as a catalyst was refluxed in DCM; excellent amounts of products were obtained. The reusability of recovered catalyst was mentioned.

Hasaninejad et al. (2011) demonstrated use of sulfuric acid-modified PEG-6000 (PEG-OSO_3_H, Table 1) as a catalyst for the microwave irradiated Friedlander annulation under solvent free conditions [54]. Well ground mixture of Friedlander components including PEG-OSO_3_H was subjected to irradiation in microwave oven at 600W; excellent amounts of substituted quinolines were obtained. The recyclability of the recovered catalyst for many subsequent cycles was found. The method reported excellent yields under solvent free conditions as well as an efficient reusability of the catalyst. 

Nasseri et al. (2013) demonstrated Friedlander synthesis using PEG-SO_3_H (Table 1) as a catalyst in aqueous medium. The aqueous mixture of 2-amino-5-chlorobenzophenone or 2-aminobenzophen- one, active methylene compounds and PEG-SO_3_H was stirred at 60 °C; affording good to excellent yields of substituted quinolines [55]. The recovered catalyst was reused for six subsequent cycles with slight decrease in activity after each rerun. Variety of active methylene compounds were used, both in cyclic and acyclic forms thereby contributed a rich library of quinoline scaffolds to the literature. The use of green solvent, short reaction times, economic yields, simple work up and reusability of catalyst makes the protocol remarkable for the improvements in the Friedlander reaction.

Zakerinasab et al. (2015) achieved Friedlander synthesis using niobium-(V) chloride /- polyethylene glycol (NbCl_5_^.^PEG, Table 1) and niobium (V) chloride (NbCl_5_) as a catalyst in glycerol as a solvent [56]. Good to excellent amounts of yields were reported. The reusability of recovered catalyst was mentioned. Both the catalyst NbCl_5_^.^PEG and NbCl_5 _proved noticeably efficient. Although the methodology claimed greener approach but use of solvent at higher temperature and usual work up procedure makes it align to conventional and limits the credit.

#### 3.1.5. Cyclodextrin as catalyst

**Scheme 8:**
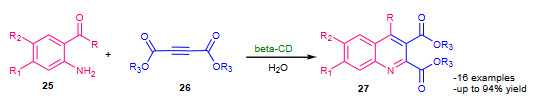
β-CD catalyzed modified Friedlander reaction.

Cyclodextrins are able to form host-guest complexes with hydrophobic molecules. Different forms of attractive forces like van der Waals interactions are believed to involve in the formation of inclusion complex between guest molecule and CDs [57]. Madhav et al. (2010) explored modified Friedlander synthesis using supramolecular catalyst β-CD (Table 1) in aqueous medium [58]. The aqueous reaction mixture containing substituted 2-aminobenzophenone (**25**), dialkyl acetylenedicarboxylate (**26**) and catalyst β-CD was heated, in which excellent yields of polysubstituted quinolines (**27**) were obtained (Scheme 8). The reusability of recovered catalyst for four consecutive cycles with slight decrease in activity after each re-run was mentioned. The protocol employed greener approach and seems conventional with solvent extraction work up procedure.

### 3.2. Inorganic polymers

#### 3.2.1.Silica as catalyst and catalyst supports

Desai et al. (2006) applied silica gel supported sodium hydrogen sulphate (NaHSO_4_-SiO_2, _Table 2) as a heterogeneous catalyst for the synthesis of 1,2,3-trisubstituted quinolines via solvent free Friedlander annulation [59]. Excellent amounts of yields were reported. The efficient reusability of recovered catalyst for three successive runs was reported. New catalyst system has been added to the advancements in the Friedlander reaction although the method involved longer reaction times.

**Table 2:** Inorganic polymers-based catalysts for optimized Friedlander reaction.

Types of inorganic polymer	Polymer supported catalysts	Amount of catalyst(ag, bmol %, c wt %)	Solvent	Energy source	Temp.(rt = room temp.)	Time(d h, e min, f s)	Yield(%)
Silica	NaHSO4-SiO2	0.2a	-	Heat	70 °C	3d	95
					100 °C	1d	92
	SSA	0.16a	-	Heat	100 °C	45e	95
		0.04a	-	MW	-	5e	85
	p-TSA/SiO2	0.26/0.5a	-		-	12e	>95
	HClO4-SiO2	1a	MeCN	Heat	60 °C	2d	96
	I2-SiO2	0.05/0.1a	-		60 °C	2d	80
	PMA-SiO2	0.1a	Ethanol		Reflux	75e	88
	Alum-SiO2	25b	-		30 °C	1.5d	99
	P2O5/ SiO2	0.4a	-		80 °C	15e	93
	HCl.SiO2	0.8a	-	MW	-	0.5d	51
	SiO2-Cl	0.1a	-	Ultrasonication	28–30 °C	1d	80
Clays & minerals	Montmorillonite KSF	1a	-	MW	-	4e	82
	Ru/HT-N	0.3a	Toluene	Heat	100 °C	20d	87
	Zeolites (H-FAU)	0.1a			90 °C	6d	73
	MCM-41-SO3H	0.015a	-	-	rt	15e	93
	MK-10	0.1a	-	MW	115 °C	4e	91
Heteropoly-acidand ions	Ag3PW12O40	20b	Ethanol	Heat	Reflux	3d	92
	H3PW12O40	1b	-		80 °C	2d	93
		3.47b			100 °C	1d	73
	H6 [P2W18O62]	20b		Heat	70 °C	2d	92
	Cs2.5H0.5PW12O40	0.2a			100 °C	15e	91
	PW/SiO2PW/KSFPW/C	40c			100 °C	15e10e15e	989694
PPA	PPA	2–3a	-		70 °C	5d	79
		0.2a	PEG	Heat MW	120 °C	24d30e	7889
Alumina	Basic Al2O3	1b	-	Grinding	rt	8e	88

Shaabani et al. (2005) employed silica sulfuric acid (SSA, Table 2) as a solid acid catalyst under solvent free conditions for Friedlander reaction [60]. Simple work up, economic yields, solvent free condition, short reaction times and reusability of SSA catalyst highlighted the valuable improvement in the Friedlander reaction.

**Scheme 9:**
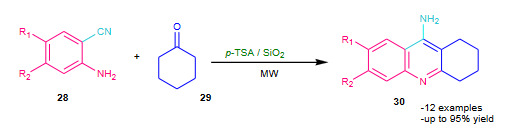
p-TSA/SiO2 catalysed modified Friedlander reaction.

Khalilzadeh et al. (2007) attempted microwave assisted solvent free Friedlander quinoline synthesis using *p*-toluenesulfonic acid supported on silica gel (*p*-TSA/SiO_2, _Table 2) as a heterogeneous catalyst [61]. The anthranilonitrile (**28**) and cyclohexanone (**29**) was reacted in the presence of the catalyst *p*-TSA/SiO_2_ under solvent free conditions; affording good yields of substituted 9-amino-1,2,3,4- tetrahydro-acridines (**30**) (Scheme 9). Variety of solid supports were screened to optimize of the catalytic activity such as acidic alumina, zeolite (HY), montmorillonite (K-10) and silica gel; among them silica found most suitable. The *p*-TSA/SiO_2 _catalyst system was evaluated under both conventional and microwave conditions; microwave irradiated protocol was found to be comparatively more efficient.

Narasimhulu et al. (2007) investigated silica supported perchloric acid (HClO_4_-SiO_2, _Table 2) as catalyst for the synthesis of quinoline derivatives by Friedlander heteroannulation [62]. Acetonitrile was used as solvent, and the method provided excellent yields. Recovered catalyst reused for three cycles. Albeit, the discussed HClO_4_-SiO_2 _catalyst approach seems conventional, it has contributed new efficient catalyst to the advancements in the Friedlander synthesis. 

Zolfigol et al. (2007) evaluated molecular iodine supported on silica gel (I_2_-SiO_2, _Table 2) as a heterogeneous catalyst for the solvent free Friedlander heteroannulation [63]. The methodology was found to be added a new form of silica supported catalyst but seems slightly less impressive due to average to good yields and lack of reusability of catalyst but the solvent free reaction at low temperature conditions marks it entry to the catalyst library for the advancement of Friedlander reaction. 

Dabiri et al. (2007) described improved Friedlander synthesis using sodium hydrogen sulphate (NaHSO_4_-SiO_2, _Table 2) under solvent free conditions at higher temperature compared to earlier reported method [64]. Excellent yields of substituted quinolines were obtained in short reaction times. The efficiency recovered catalyst was retained for three consecutive cycles.

Das et al. (2008) attempted conventional Friedlander synthesis using silica supported phosphomolybdic acid (PMA-SiO_2, _Table 2) as catalyst [65]. The ethanolic reaction mixture containing Friedlander components was refluxed in the presence of PMA-SiO_2 _catalyst; affording good amounts of products. The reusability of catalyst was mentioned without frequency and efficiency. 

Mohammadi et al. (2008) reported solvent free synthesis of polysubstituted quinolines using KAl(SO_4_)_2_·12H_2_O-SiO_2_ (Alum-SiO_2, _Table 2) as a catalyst [66]. Variety of active methylene compounds were used for the synthesis; furnished excellent amounts of corresponding quinolines. The recovered catalyst was recycled for six subsequent runs with negligible loss of activity after each run. The Alum-SiO_2_ catalyzed protocol seems to be attractive due to solvent free and low temperature conditions along with excellent yields and reusability of the catalyst.

Zolfigol et al. (2008) demonstrated microwave assisted solvent free Friedlander synthesis using silica sulfuric acid (SSA, Table 2) as a heterogeneous catalyst [67]. The neat Friedlander reaction mixture including SSA as a catalyst was microwave irradiated (900W); furnished good to excellent amounts of substituted quinolines. The solvent free condition and shorter reaction times makes the method remarkable. 

Hasaninejad et al. (2011) reported silica-supported P_2_O_5_ (P_2_O_5_/ SiO_2, _Table 2) as a catalyst for solvent-free synthesis of poly-substituted quinolines via Friedlander heteroannulation reaction [68]. Good to high amount of quinolines were reported. Solvent free condition and short reaction times makes the method attractive.

**Scheme 10:**
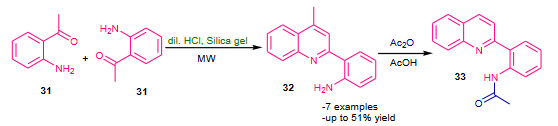
HCl.SiO2 catalyzed dimerization reaction.

Al-Qahtani et al. (2013) proposed microwave assisted synthesis of polysubstituted quinolines by Friedlander reaction using dilute hydrochloric acid and silica gel (HCl.SiO_2, _Table 2) as catalyst [69]. The mixture of 2-aminoacetophenone (**31**), one drop of diluted hydrochloric acid and silica gel was subjected to microwave irradiation at 440 watts for 0.5 h; affording dimerization product **32** (Scheme 10). The same methodology was employed for the reaction between **31** and substituted 2-hydroxyacetophenone; average yields of corresponding quinolines were reported.

Sudha et al. (2013) investigated the use of silica chloride (SiO_2_-Cl, Table 2) for the preparation of polyfunctional quinoline derivatives under solvent free conditions and ultrasonication [70]. The Friedlander reaction components added with SiO_2_-Cl catalyst was sonicated neatly in a SIDILU sonicator (35 KHz and 120 W); furnished good yields of substituted quinolines. The reusability of catalyst was reported for two subsequent cycles with continuous decrease in activity. The protocol added new form of silica-based catalyst to the advancement in the Friedlander reaction. In spite of less economic yields the method seems attractive due to use of solvent free and ultrasonication conditions. 

#### 3.2.2. Clays and minerals as catalyst and catalyst supports

Sabitha et al. (1999) attempted microwave assisted solvent free synthesis of polycyclic quinoline derivatives using montmorillonite KSF clay (Table 2) as a catalyst [71]. The solvent free reaction mixture containing Friedlander substrates and KSF clay catalyst was microwave irradiated (600W); affording considerable amounts of corresponding quinolines. Typical active methylene compounds were used to prepare polycyclic quinoline derivatives. In spite of less economic yields; the solvent free condition, short reaction times and addition of new quinoline scaffolds to the quinoline library makes the methodology creditable.

**Scheme 11:**
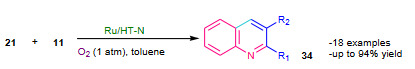
Ru/HT-N catalyzed modified Friedlander reaction.

Motokura et al. (2004) demonstrated one pot two step synthesis of quinolines using Ru-grafted hydrotalcite (Ru/HT, Table 2) as a catalyst via modified Friedlander reaction [72]. The catalyst Ru/HT was prepared in the presence of triethylamine and labelled Ru/HT-N using RuCl_3_.nH_2_O, hydrotalcite (HT), Mg_6_Al_2_(OH)_16_CO_3_ and triethylamine. The variety of catalyst systems were screened for optimization of the reaction such as HT, Ru/Al_2_O_3_, Ru/MgO, Ru/ Mg(OH)_2_, and Ru/Al(OH)_3_ among them, Ru/HT-N (Table 2) proved to be most efficient. The reaction mixture of 2-aminobenzyl alcohol (**21**), active methylene compounds (**11**) and catalyst Ru/HT-N was vigorously stirred at 100 °C in toluene under an O_2 _atmosphere for 20 h; affording good amounts of corresponding quinolines (**34**) (Scheme 11). Reusability of recovered catalyst was mentioned with consistent catalytic activity. Although the protocol seems conventional and less impressive due use of toxic solvent and longer reaction times, it has contributed another modified approach aligns to the Friedlander heteroannulation. 

López-Sanz et al. (2010) reported zeolites (HY) as a catalyst for Friedlander quinoline synthesis. The zeolites H-BEA, H-MFI, H-FAU and H-MOR were investigated for catalytic performance; among them H-BEA and H-FAU (Table 2) were found to be the most efficient catalysts for the quinoline synthesis [73]. The yields were average to good. Under both the conditions (using toluene as solvent and solvent free), the Friedlander reactions were performed in the presence of zeolite catalyst; the solvent free protocol found more efficient**.**

Maleki et al. (2014) proposed Friedlander annulation using silica based sulfonic acid (MCM-41-SO_3_H, Table 2) as an aluminosilicate mineral based catalyst under solvent free conditions [74]. Excellent amounts of quinoline derivatives were obtained in short reaction times. The reusability of catalyst MCM-41-SO_3_H was mentioned for several consecutive cycles without considerable loss in activity. The protocol found to be added an efficient catalyst to the advancement in the Friedlander reaction.

**Scheme 12:**
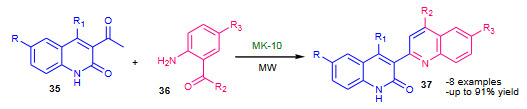
MK-10 catalyzed synthesis of quinolinyl- quinolinones.

Subashini et al. (2014) reported microwave assisted solvent free Friedlander synthesis of quinolinyl quinolinones by using montmorillonite K-10 (MK-10, Table 2) as a heterogeneous catalyst [75] (Scheme 12). The synthesis of quinolinyl quinolinones was performed under conventional as well as microwave irradiated conditions. The conventional synthesis employed use of conc. H_2_SO_4_ in glacial acetic acid while microwave (power 500W) assisted protocol used MK-10 catalyst under solvent free conditions. The later solvent free protocol proved to be most efficient compare to conventional approach by showing excellent yields in shorter reaction times.

#### 3.2.3. Heteropolyacid and anions as catalyst and supports

Yadav et al. (2004) utilized silver phosphotungstic acid (Ag_3_PW_12_O_40, _Table 2) as a heteropoly acid-based catalyst for the Friedlander quinoline synthesis [76]. The ethanolic reaction mixture containing Friedlander components was refluxed in the presence of Ag_3_PW_12_O_40_ catalyst; affording good amount of yields of substituted quinolines. Varieties of active methylene compounds were used. The recovered catalyst was recycled after activation for four consecutive cycles with gradual decrease in activity after each rerun. Although the protocol seems conventional with use of solvent and longer reaction times; it has successfully added the new form of catalyst system to the improvement in the Friedlander reaction. 

Dabiri et al. (2009) attempted solvent free Friedlander synthesis using phosphotungstic acid (H_3_PW_12_O_40, _Table 2) as a heterogeneous catalyst [77]. Different active methylene compounds were used to prepare 2,3,4-trisubstituted quinolines. Excellent amounts of desired quinolines were reported. The reusability of the recovered catalyst was mentioned for five subsequent runs with almost consistent catalytic activity. The solvent free condition, efficient reusability of catalyst and excellent yields has highlighted the noticeable advantages of the discussed method.

Heravi et al. (2010) evaluated role of Wells–Dawson type of heteropoly acid H_6_ [P_2_W_18_O_62_] (Table 2) as a solid acid catalyst for the synthesis of quinolines via Friedlander reaction under solvent free conditions [78]. The catalytic activity of heteropolyacids such a H_6_[P_2_W_18_O_62_], H_5_[PMo_10_V_2_O_40_] and H_6_[PMo_9_V_3_O_40_] were screened for catalytic performance; H_6_ [P_2_W_18_O_62_] proved most efficient with excellent yields. The reusability of catalyst for five consecutive cycles was mentioned with gradual decrease in activity after each cycle. 

Rafiee et al. (2011) employed cesium partially substituted phosphotungstate (Cs_2.5_H_0.5_PW_12_O_40, _Table 2), a heteropoly salt as a catalyst for the Friedlander reaction under solvent free conditions [79]. The acidic salts of Cs_x_H_3-x_PW_12_O40 (x = 1.0, 2.0 and 2.5) were prepared using literature method; among all the Cs_2.5_H_0.5_PW_12_O_40_ salt found to be more efficient catalyst. The solvent free condition, excellent yields, short reaction times and reusability of the catalyst make the method advantageous over conventional approaches. The same group has reported Keggin type heteropoly acids (HPAs) and supported ones on solids with different nature and textural properties as a catalyst under solvent free conditions for Friedlander synthesis [80]. The tungstophosphoric acid (H_3_PW_12_O_40_, PW) supported on silica (PW/SiO_2_), KSF (PW/KSF), activated carbon (PW/C) and γ-alumina (PW/γ-Al_2_O_3_) (Table 2); among these all the catalyst system found to be equally efficient except PW/γ-Al_2_O_3_. Reusability of all the catalyst systems was reported for four consecutive cycles with gradual decrease in activity after each cycle. The article contributed efficient catalyst systems to the advancement of Friedlander reaction. The solvent free condition, excellent yields, shorter reaction times, reusability of catalyst seems creditable.

**Scheme 13:**
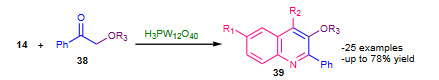
H3PW12O40 catalyzed synthesis of heteroatom substituted quinolines.

Chen et al. (2014) investigated use of phosphotungstic acid (H_3_PW_12_O_40, _Table 2) as a catalyst for the Friedlander synthesis of novel heteroatom substituted quinolines under solvent free conditions [81]. The substituted 2-aminoaryl ketones (**14**) and a heteroatom bearing ketones (**38**) was heated solvent free in the presence of H_3_PW_12_O_40_ catalyst; affording corresponding quinoline scaffolds (**39**) (Scheme 13**)**. The recovered catalyst was reused for five subsequent cycles with slight decrease in activity up to third cycle and thereafter it decreases considerably. Although the products were average to good, the authors have reported a rich library of heteroatom substituted quinoline scaffolds which make the valuable contribution.

#### 3.2.4. Polyphosphoric acid (PPA) as catalyst

**Scheme 14:**
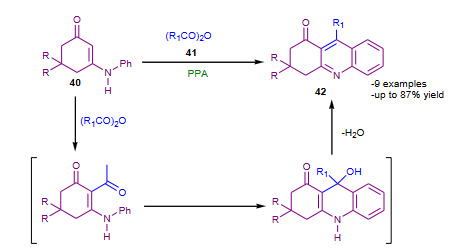
PPA mediated modified Friedlander synthesis.

Na et al. (2005) demonstrated modified Friedlander synthesis of quinolines in the presence of PPA (Table 2) [82]. The substituted enaminones (**40**) were treated with different anhydrides (**41**) PPA; affording corresponding quinolines (**42**) (Scheme 14). Though yields of quinolines were average to good, but the protocol has proposed new route for synthesis of quinolines with a modified Friedlander approach.

**Scheme 15:**
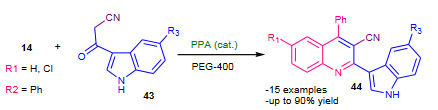
PPA catalysed modified Friedlander synthesis.

Shiri et al. (2012) reported synthesis 2-(indol-3-yl)-3-nitriloquinolines using PPA (Table 2) as a catalyst via modified Friedlander heteroannulation under thermal as well as microwave assisted conditions [83]. The mixture of substituted amino ketones (**14**), substituted 3-cyanoacetylindoles (**43**) and catalyst PPA in PEG-400 was subjected to thermal or microwave irradiation (350W); furnished good yields of corresponding 2-(indol-3-yl)-3-nitriloquinolines (**44**) (Scheme 15). Compare to thermal method, microwave protocol found to be most efficient with shorter reaction times and higher yields. The use of green solvent and contribution of diverse quinoline scaffolds to the library highlighted the valuable advancement to the Friedlander annulation.

#### 3.2.5. Alumina as catalyst

**Scheme 16:**
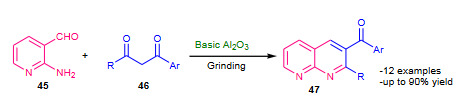
Alumina catalyzed modified Friedlander synthesis.

Mogilaiah and Vidya (2007) proposed Friedlander synthesis of substituted 1, 8-naphthyridines in the solid state using basic alumina (Al_2_O_3, _Table 2) as a catalyst [84]. The mixture of 2-aminonicotinaldehyde (**45**), active methylene compound (**46**) and basic Al_2_O_3_ was ground by pestle and mortar at room temperature; affording excellent yields of 1, 8-naphthyridines (**47**) (Scheme 16). Short reaction times, excellent yields and use of mortar-pestle provide the creditable improvement in the Friedlander reaction.

### 3.3. Hybrid polymers

#### 3.3.1. Organosilica based hybrid polymers as catalyst

Domínguez-Fernández et al. (2009) demonstrated Friedlander quinoline synthesis using basic mesoporous amino-grafted MCM-41 as hybrid catalysts [85]. The variety of MCM-41 materials supporting aminopropyl (AP), methylaminopropyl (MAP), and diethylaminopropyl (DEAP) groups were prepared and also modified the acidic properties of Al-SBA-15 by incorporating cesium ions. Among all the catalyst systems, DEAP-MCM-41 and MAP-MCM-41 (Table 3) catalysts found most efficient with toluene. Although use of toxic solvent and conventional approach limits the scope of method, but the variety of catalysts systems that have been investigated for the Friedlander reaction added catalyst variations.

**Table 3:** Hybrid polymers-based catalysts for optimized Friedlander reaction.

Type of hybrid polymer	Polymer supported catalysts	Amount of catalyst(ag, bmol %, c wt %)	Solvent	Energy source	Temp.(rt = room temp.)	Time(dh, e min, f s)	Yield(%)
Organosilica	DEAP-MCM-41MAP-MCM-41	20c	Toluene	Heat	100 °C	2d1d	93100
	PSS	0.1a	-		80 °C	5d	17
				MW	80 °C	30e	90
	SBA-15/APS	0.025a	Toluene	Heat	90 °C	3d	86
	AP/Al-MCF	0.05a	-		50 °C	5d	71
	Perfluoroalkylsulfonic acid functionalized surface	-	MeCN		70 °C	13e	90
	2APMS/NbMCF	0.025a	-		50 °C	30e	98
	Silica-supported binam-S-proline sulphonamide	20b			25 °C	24d	93
Metal-organic framework (MOF)	Cu3(BTC)2(Basolite C300)	0.05a	-		80 °C	2d	96
			DCM		120 °C	2d	92
	CuBDC	3b	DMF		80 °C	20e	99
Miscellaneous	T3P	50b	-		60 °C	30e	95
				MW	100 °C	5e	95
	TPAPol60	0.1a	Ethanol	Heat	Reflux	24d	98

**Figure 5:**
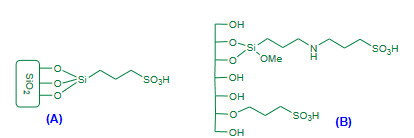
(A) PSS catalyst; (B) SBA-15/APS catalyst.

Garella et al. (2009) proposed solvent free microwave assisted Friedlander heteroannulation using propylsulfonic acid bounded silica (PSS, Figure 5A, Table 3) as a catalyst [86]. The catalyst activity was performed under both the microwave (200W) as well as thermal conditions; microwave assisted protocol proved most efficient with shorter reaction times and higher yields. Varied yields of polysubstituted quinolines were reported. The catalyst recyclability was described for two more subsequent cycles without appreciable loss in catalytic activity.

López-Sanz et al. (2012) reported inorganic-organic hybrid materials based on SBA-15 molecular sieves as a catalyst for the Friedlander annulation [87]. Different catalyst systems based on SBA-15 were evaluated in toluene among them trialkoxysilyl 3-(propylamino) propane-1-sulfonic acid bounded on SBA-15 (SBA-15/APS, Figure 5(B), Table 3) found most efficient. The article contributed new genre of hybrid catalyst although it involved lengthy catalyst preparation steps, use of toxic solvent.

Smuszkiewicza et al. (2013) reported bifunctional mesoporous MCF materials as catalysts in the Friedlander condensation under solvent free conditions [88]. The MCF materials modified with (3-aminopropyl)trimethoxysilane; the silica MCF (AP/MCF), nio-biosilicate Nb-MCF (AP/Nb-MCF) and aluminosilicate Al-MCF (AP/Al-MCF, Table 3) were used as catalyst for Friedlander reaction. Among these, AP/Al-MCF seems comparatively most efficient with shorter reaction time and higher yields. The reusability of catalyst was also mentioned. The methodology shared new form of hybrid catalysts to the Friedlander synthesis. The same group has been reported the nio-biosilicate Nb-MCF with [3-(2-aminoethylamino) propyl] trimethoxysilane (2APMS), noted as 2APMS/NbMCF. Along with 2APMS/NbMCF, AP/MCF both in activated and nonactivated form were used as a catalyst. Nonactivated samples were found to be more efficient catalysts than the activated form [89].

**Figure 6:**
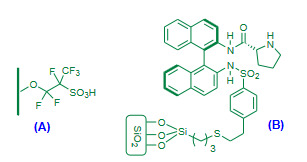
(A) Sulfonic acid functionalized microreactor surface as a catalyst; (B) Silica-supported binam-S-proline sulphonamide hybrid catalyst.

Ricciardi et al. (2013) demonstrated perfluoroalkylsulfonic acid monolayer-functionalized microreactor as a heterogeneous catalyst (Figure 6A, Table 3) for the Friedlander annulation [90]. The 2-aminoacetophenone and ethyl acetoacetate in dry acetonitrile were passed through the continuous flow microreactor; affording excellent yields. The method added an innovative small-scale approach for the Friedlander reaction which involved steady inner acid functionalized surface of microreactor as a catalyst.

Bañón-Caballero et al. (2013) investigated solvent free enantioselective Friedlander heteroannulation using silica-supported binam-*S*-proline sulphonamide (Figure 6B, Table 3) as a novel hybrid heterogeneous catalyst [91]. The procedure involved use of water along with 2-aminobenzaldehyde, corresponding ketone and the hybrid catalyst. The recyclability for four consecutive cycles was mentioned with almost negligible loss in catalytic activity and enantioselectivity. 

#### 3.3.2. Metal-organic frameworks based hybrid polymers as catalyst

Pérez-Mayoral and Čejka (2011) proposed Friedlander synthesis using a metal-organic framework (MOF) based hybrid materials as a catalyst under solvent free conditions [92]. The [Cu_3_(BTC)_2, _Table 3] (BTC=benzene-1,3,5-tricarboxylate) is a 3D porous MOF with a zeolite-like structure and which is commercially available as Basolite C300. Along with Basolite C300, two different molecular sieves, such as H-BEA and the mesoporous material (Al)SBA-15 were also employed as a catalyst. [Cu_3_(BTC)_2_] showed highly improved catalytic activity as compared with the molecular sieves, H-BEA and (Al)SBA-15. The same research group has reported one more paper using CuBTC MOF as efficient catalyst [93]. The article added new version of hybrid catalyst to the advancement in the Friedlander annulation. 

Phan et al. (2013) attempted modified Friedlander quinoline synthesis using CuBDC (BDC= 1,4-benzenedicarboxylate) as a MOF based hybrid heterogeneous catalyst [94]. The modified Friedlander reaction involved use of 2-aminobenzylalcohol with corresponding ketone was treated in the presence of CuBDC (Table 3) catalyst; high amount of yield obtained. The recovered catalyst was reused for several times without significant loss in catalytic activity. Although, the MOF catalyst proved itself as efficient but, its preparation and characterization seem costlier.

#### 3.3.3. Miscellaneous hybrid polymers as catalyst

**Figure 7:**
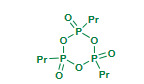
T3P as a hybrid catalyst.

Jida and Deprez (2012) attempted Friedlander synthesis of polysubstituted quinolines and naphthyridines using propylphosphonic anhydride (T3P, Figure 7, Table 3) as a catalyst [95]. The reaction involved use of T3P (50% in ethyl acetate) as a promoter, after the completion of reaction water was added to the reaction mixture which dissolves the T3P and provides simple work up procedure. Excellent yields of substituted quinolines and naphthyridines were mentioned. The article has been successfully added a new form of catalyst with rich library of quinoline scaffolds.

Bennardi et al. (2015) employed tungstophosphoric acid included in a polymeric matrix of polyacrylamide (TPA_Pol60_, Table 3) as a hybrid catalyst using Friedlander heteroannulation [96]. The ethanolic mixture of Friedlander reaction including TPA_Pol60_ catalyst was refluxed; affording excellent yields of corresponding quinolines. The catalyst reusability was mentioned with consistent catalytic activity. Although the methodology seems conventional with longer reaction times; it has been found to be added a new form of hybrid catalyst to the improvement in the Friedlander reaction. 

### 3.4. Biocatalysts

Shaabani et al. (2008) demonstrated solvent free Friedlander quinoline synthesis using biodegradable sulfonated cellulose and starch as a solid acid catalyst [97]. Both the sulfonated cellulose and starch proved as an efficient biocatalyst, furnished good to excellent amounts of products but sulfonated cellulose (Table 4) found comparatively more efficient with shorter reaction times and higher yields. The article introduced new natural polymer supported catalyst for the advancement in the Friedlander annulation. The article limits the credit with lack of reusability of catalyst and high temperature conditions.

**Table 4:** Polymeric biocatalysts for optimized Friedlander reaction.

Type of biocatalyst	Polymer supported catalysts	Amount of catalyst(ag, bmol %, cwt %)	Solvent	Energy source	Temp.(rt = room temp.)	Time(d h, e min, f s)	Yield(%)
Cellulose	Cellulose-SO3H	0.08a	-	Heat	100 °C	30e	85
Polysacc-harides	Chitosan	20b	-		80 °C	2e	89
			Methanol		Reflux	5d	75
	Chitosan-SO3H	0.1a	Ethanol		Reflux	20e	90

Siddiqui and Khan (2013) achieved solvent free synthesis of benzopyranopyridines using chitosan (Table 4) as a biodegradable, heterogeneous catalyst via Friedlander cyclocondensation [98]. The catalyst activity was evaluated under both in refluxing methanol and solvent free conditions; solvent free conditions gave higher yields in shorter reaction times. The reusability of the catalyst for five consecutive cycles was mentioned with negligible loss in activity. Considerably short reaction times, solvent free condition, high yields, efficient reusability of catalyst and environment friendly catalyst makes the impressive contribution to the Friedlander advancements. 

Reddy et al. (2013) investigated synthesis of quinoline by Friedlander approach using chitosan sulfonic acid (chitosan-SO_3_H, Table 4) as a biodegradable solid acid catalyst [99]. The ethanolic reaction mixture of Friedlander components including chitosan-SO_3_H catalyst was reflux; affording high to excellent yields of products. The recovered catalyst was recycled for three subsequent cycles with negligible loss in catalytic activity. Although the methodology seems conventional but the short reaction times, efficient reusability of catalyst and use of biodegradable catalyst make the creditable improvement to the Friedlander condensation.

### 3.5. Nanocatalysts

#### 3.5.1. Magnetic nanocatalysts

##### 3.5.1.1. Silica based magnetic nanocatalysts

**Figure 8:**
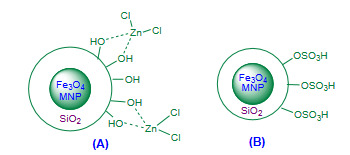
(A) Fe3O4@SiO2/ZnCl2 nanocatalyst; (B) Fe3O4@SiO2-SO3H nanocatalyst.

Soleimani et al. (2017) reported ZnCl_2_ supported on silica-coated magnetic nanoparticles of Fe_3_O_4_ (Fe_3_O_4_@SiO_2_/ZnCl_2_, Figure 8A, Table 5) as a magnetically recoverable core-shell nanocatalyst for the Friedlander quinoline synthesis under solvent free conditions [100]. Excellent yields of polysubstituted quinolines were obtained. The recovered Fe_3_O_4_@SiO_2_/ZnCl_2 _nanocatalyst was recycled for five consecutive cycles with consisted activity up to second run and thereafter it decreases considerably. Economic yields, solvent free and low temperature conditions, magnetic recovery of catalyst, reusability of catalyst seems the valuable contribution of method; though it involved multistep preparation of catalyst.

**Table 5:** Magnetic nanocomposites-based catalyst for optimized Friedlander reaction.

Type of magnetic nanocomposite	Polymer supported catalysts	Amount of catalyst(ag, bmol %, cwt %)	Solvent	Energy source	Temp.(rt = room temp.)	Time(d h, e min, f s)	Yield(%)
Silica based magnetic nanocatalysts	Fe3O4@SiO2/ZnCl2	0.07a	-	Heat	80 °C	2d	96
	Fe3O4@SiO2-SO3H	0.05a			110 °C	1.75d	94
Hybrid magnetic nanocatalysts	γ-Fe2O3-HAp-(CH2)3-NHSO3H	0.7b			rt	3d	93
	Fe3O4@SiO2@PDETSA	0.02a			110 °C	45e	98
	Fe3O4@SiO2-APTES-TFA	0.2a			100 °C	5d	98
	Fe3O4@SiO2-imid-PMAn	0.02a			70 °C	50e	95
	Fe3O4-Cys	0.1a	-	MW	-	3e	93
Miscellaneous	Nanocat-Fe-OSO3H	0.05a	-		130 °C	4d	92
	CuFe2O4	5b	Water	Heat	80 °C	32e	95
	ZrO2/Fe3O4-MNPs	0.02a	Ethanol		70 °C	25e	92

Beyki and Fallah-Mehrjardi (2017) demonstrated solvent free Friedlander synthesis of polysubstituted quinolines using Fe_3_O_4_@SiO_2_-SO_3_H (Figure 8B, Table 5) as a recyclable heterogeneous nanomagnetic catalyst [101]. Variety of active methylene compounds were used; affording high to excellent amounts of corresponding quinolines. The magnetically recovered nanocatalyst was reused for five subsequent cycles with gradual decrease in catalytic activity after each rerun. The catalyst efficiency was compared with earlier reported protocols. Although the method involves high temperature condition, but high yields, solvent free conditions, simple recovery and efficient reusability of catalyst seems advantageous.

##### 3.5.1.2. Hybrid magnetic nanocatalysts

**Figure 9:**
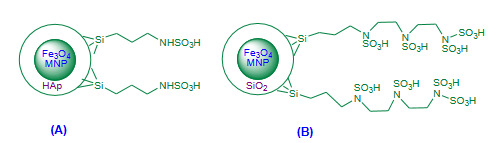
(A) γ-Fe2O3-HAp-(CH2)3-NHSO3H nanocatalyst; (B) Fe3O4@SiO2@PDETSA nanocatalyst.

Sheykhan et al. (2011) [102] reported solvent free room temperature Friedlander heteroannulation using sulfamic acid heterogenized on hydroxyapatite encapsulated γ-Fe_2_O_3_ nanoparticles as a magnetic catalyst [γ-Fe_2_O_3_-HAp-(CH_2_)_3_-NHSO_3_H, Figure 9A, Table 5]. The catalyst preparation involved sequence of steps. Excellent amounts of products and reusability of nanocatalyst for several ten more consecutive cycles without appreciable loss in activity was reported. The efficiency of [γ-Fe_2_O_3_-HAp-(CH_2_)_3_-NHSO_3_H] nanocatalyst at the room temperature found remarkable part of the protocol. 

Nasseri et al. (2014) attempted solvent free Friedlander heteroannulation using Fe_3_O_4_@SiO_2 _bonded *N*-propyl diethylene tetrasulfamic acid (Fe_3_O_4_@SiO_2_@PDETSA, Figure 9B, Table 5) as a super paramagnetic nanocatalyst [103]. The method provided good to excellent amounts of corresponding quinolines. The recovered catalyst was reused for eight subsequent cycles with negligible loss in activity after each rerun. The protocol has been found to be added a novel magnetic nanocatalyst successfully, but the preparation of catalyst involved many steps which limits the credit of the methodology.

**Figure 10:**
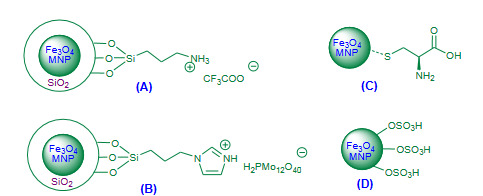
(A) Fe3O4@SiO2-APTES-TFA nanocatalyst; (B) Fe3O4@SiO2-imid-PMAn nanocatalyst; (C) Fe3O4-Cys nanocatalyst; (D) Fe3O4-OSO3H nanocatalyst.

Jafarzadeh et al. (2015) validated trifluoroacetic acid-aminopropyl triethoxysilane immobilized on silica coated magnetite nanoparticles (Fe_3_O_4_@SiO_2_-APTES-TFA, Figure 10A, Table 5) as a magnetic nanocatalyst for the solvent free Friedlander quinoline synthesis [104]. Excellent yields of polysubstituted quinolines were reported. Magnetically recovered catalyst was reused for four subsequent runs without significant decrease in the catalytic activity. The article introduced new hybrid magnetic nanocatalyst with efficient catalytic performance though it involved multistep preparation of catalyst and longer reaction times.

Esmaeilpour and Javidi (2015) demonstrated synthesis of polysubstituted quinolines via Friedlander heteroannulation using Fe_3_O_4_@SiO_2_-imid-PMA nanoparticles as a magnetic nanocatalyst under solvent free conditions [105]. The heteropolyacid phosphomolybdic acid (PMA, H_3_PMo_12_O_40_) was immobilized in both the form nano H_3_PMo_12_O_40 _(PMA^n^), and bulk H_3_PMo_12_O_40 _(PMA^b^) on the Fe_3_O_4_@SiO_2_-imid nanoparticles; both the form of catalysts showed excellent efficiency but Fe_3_O_4_@SiO_2_-imid-PMA^n^ (Figure 10B, Table 5) found more efficient with higher yields. The recovered nanocatalyst was recycled reused for four times without noticeable decrease in catalytic activity. The method added a new hybrid nanocatalyst though it involved lengthy preparation of catalyst.

Bankar and Shelke (2018) reported microwave assisted Friedlander synthesis using ferrite-L-cysteine (Fe_3_O_4_-Cys, Figure 10C, Table 5**)** as a magnetic nanocatalyst under solvent free conditions [106]. The efficiency of Fe_3_O_4_-Cys nanocatalyst was compared to the earlier reported catalysts protocol. The methodology added a new efficient hybrid magnetic nanocatalyst to the Friedlander advancement with short reaction times, excellent yields and recyclability of the catalyst for ten consecutive cycles was mentioned with no significant loss in catalytic activity.

##### 3.5.1.3. Miscellaneous magnetic nanocatalysts

Gawande et al. (2013) reported microwave assisted Friedlander heteroannulation using magnetite-sulfonic acid nanoparticles (Nanocat-Fe-OSO_3_H, Figure 10D, Table 5) as a magnetic heterogeneous nanocatalyst under solvent free conditions [107]. Along with Friedlander reaction, the article also described the use of Nanocat-Fe-OSO_3_H as a catalyst for Ritter reaction and a multicomponent reaction. The model reaction was performed using Nanocat-Fe-OSO_3_H as a catalyst in short time under microwave irradiation; affording high yield. The reusability of magnetically recovered catalyst was mentioned for five subsequent cycles with negligible loss in catalytic activity. 

Baghbanian and Farhan (2014) achieved synthesis of quinolines via Friedlander reaction using CuFe_2_O_4_ nanoparticles (Table 5) as a nanocatalyst in aqueous medium [108]. The excellent yields of corresponding quinolines were obtained in short times. The recovered catalyst was reused for five successive cycles without any significant loss of activity. New form of efficient magnetic nanocatalyst was introduced to the catalyst library for Friedlander improvements. 

Hejazi et al. (2015) investigated Friedlander quinoline synthesis using zirconia supported on ferrite magnetic nanoparticles (ZrO_2_/Fe_3_O_4_-MNPs, Table 5) as a nanocatalyst in ethanolic medium [109]. The efficiency of the ZrO_2_/Fe_3_O_4_-MNPs nanocatalyst was compared with earlier reported catalysts protocols. The recovered catalyst was recycled for four consecutive cycles without noticeable loss in activity. Although the protocol seems conventional but short reaction times, excellent yields and efficient reusability of catalyst highlights the advantages of method. 

#### 3.5.2. Clay and mineral based nanocatalysts

Sadjadi et al. (2009) performed synthesis of polysubstituted quinolines via a Friedlander reaction using nanocrystalline alumina (Nano-Al_2_O_3, _Table 6) as a nanocatalyst in refluxing chloroform [110]. Excellent amounts of yields were reported. The catalyst reusability for three successive runs was mentioned with gradual decrease in activity. The methodology found to be added a new catalyst but, use of solvent, longer reaction times makes it align to conventional.

**Table 6:** Nanocomposites as catalyst for optimized Friedlander reaction.

Type of nanocomposites	Polymer supported catalysts	Amount of catalyst(ag, bmol %, cwt %)	Solvent	Energy source	Temp.(rt = roomtemp.)	Time(d h, e min, f s)	Yield(%)
Clay or mineral nanocomposites	Nano-Al2O3	3b	CHCl3	Heat	Reflux	3d	98
	Nanoporouscage-type aluminosilicate AlKIT-5	0.05a	Ethanol			2.5d	92
	Nano-SiO2	0.5a	-	MW	100 °C	6e	91
	(BSPY)HSO4/MCM-41	0.07a	-	Heat	100 °C	70e	93
	FeCl3.nano-SiO2	-	-		100 °C	-	90
	Li+ modified nanoporous Na+ -montmorillonite	0.06a	-		100 °C	1d	95
	Nano-TiO2	5b	-		80 °C	12e	92
	Nanocrystalline sulfatedzirconia (SZ)	0.05a	Ethanol		Reflux	70e	90
	Nano-CaSiO3	10b	-		120 °C	30e	93
Metal oxide nanocatalysts	Nano-CuO	5b	-		60 °C	1d	98
		3b	MeCN		40 °C	10d	93
	NF-ZnO	10b	-		100 °C	4d	98
	Nano-SnO2	5b	-	MWGrinding	rt	3e30e	82.585
Hybrid nanocatalysts	PVA/Fe(NO3)2	3b	Toluene	Heat	80 °C	4d	96
	RFCuSRFCoS(Carbon aerogel)	0.05a	-		50 °C	4d	8999
	PPInCl-nSiO2	1.8b	-		110 °C	30e	99
Miscellaneous	Nano-Pd	2b	Toluene		100 °C	20d	75
	Nano-Ag-Pd/C	1b			125 °C	21d	69
	NiNPs	10b	-		75 °C	50e	96
	RFS-PORFS-PS	0.025a	-		50 °C	15e	69

Chauhan et al. (2010) employed nanoporous cage-type aluminosilicate AlKIT-5 (Table 6) as a nanocatalyst for the synthesis of polysubstituted quinolines via Friedlander heteroannulation in refluxing ethanol [111]. The excellent yields of corresponding quinolines were reported with reusability of catalyst for three subsequent cycles with considerable decrease in catalytic activity after each cycle. The article introduced an efficient aluminosilicate mineral based nanocatalyst though the method seems conventional.

Hasaninejad et al. (2012) demonstrated solvent free microwave assisted Friedlander annulation using silica nanoparticles (Nano-SiO_2, _Table 6) as a nanocatalyst [112]. The varieties of active methylene compounds were used for quinoline synthesis; high to excellent yields were obtained. The same silica nanocatalyst was also used for synthesis of quinoxalines efficiently. The reusability for fourteen more subsequent cycles with slight decrease in activity after each rerun was mentioned.

Abdollahi-Alibeik and Pouriayevali (2012) studied application of nanosized MCM-41 supported n-butanesulfonic acid pyridinium hydrogensulfate [(BSPY)HSO_4_/MCM-41, Table 6] as a nanocatalyst for the Friedlander synthesis of quinolines under solvent free conditions [113]. The variety of active methylene compounds were used; affording high to excellent yields of substituted quinolines. The efficiency of the nano [(BSPY)HSO_4_/MCM-41] catalyst was compared with earlier reported polymeric catalysts protocol. The reusability of catalyst was unseen in the article. The solvent free reaction, short reaction times and economic yields seems the improvement over the classical Friedlander reaction.

Tahanpesar et al. (2014) reported solvent free Friedlander quinoline synthesis using ferric chloride supported on nanosilica (FeCl_3_.nano-SiO_2, _Table 6) as a nanocatalyst [114]. Good to high amount of corresponding quinolines were obtained. The paper introduced an efficient silica mineral based nanocatalyst for the advancement of Friedlander reaction.

Azimi and Abbaspour-Gilandeh (2014) demonstrated solvent free synthesis of polysubstituted/polycyclic quinolines via a solvent free Friedlander synthesis using Li^+^ modified nanoporous Na^+^-montmorillonite as a nanocatalyst [115] (Table 6). The variety of active methylene compounds were employed; furnished excellent amount of yields in short reaction times. The reusability of catalyst for five more consecutive cycles were performed and showed almost retained catalytic activity. The efficiency of catalytic activity was compared with more than 15 earlier reported catalytic protocols. 

Bandyopadhyay et al. (2014) attempted titania nanomaterials (nano-TiO_2, _Table 6) of different sizes as a heterogeneous catalyst for the Friedlander synthesis of quinolines under solvent free conditions [116]. The nano-TiO_2_ of size 16, 35, 70, 200 and 1000 nm were employed as a catalyst for the reaction; 200 and 1000 nm titania particles were inefficient as a catalyst while 16 nm size nano-TiO_2 _found the most efficient. The reusability of catalyst for four subsequent cycles was mentioned with gradual decrease in activity after each cycle. 

Teimouri and Chermahini (2016) performed Friedlander synthesis using nanocrystalline sulfated zirconia (SZ, Table 6) as a heterogeneous catalyst in refluxing ethanol [117]. Alongwith SZ, montmorillonite K-10 (K-10) and zeolite (ZMS-5) was also screened as catalyst for the reaction; all the catalyst systems proved almost equally efficient. The reusability of all the catalyst forms were reported for three successive cycles with gradual decrease in activity though SZ showed maximum efficiency. High to excellent yields of corresponding quinolines were mentioned in short reaction times.

Palaniraja et al. (2017) synthesized polysubstituted quinolines via Friedlander reaction using wollastonite (calcium silicate, CaSiO_3, _Table 6) nanoparticles as a nanocatalyst under solvent free conditions [118]. The model reaction was performed using nano-CaSiO_3 _catalyst. The article described the synthesis of calcium silicate nanoparticles using tetraethyl orthosilicate and soluble calcium nitrate tetrahydrate. The reusability of catalyst for four consecutive cycles was mentioned with gradual decrease in activity after third cycle. The library of quinolines was unseen in the article but, the article has successfully introduced a new catalyst to the advancement in the Friedlander heteroannulation.

#### 3.5.3. Metal oxide nanocatalysts

Nezhad et al. (2011) studied application of CuO nanoparticles (nano-CuO, Table 6) as a nanocatalyst for the solvent free Friedlander heteroannulation [119]. Different metal oxides such as TiO_2_, SiO_2, _Al_2_O_3_, ZnO, MgO, CuO bulk and nano-CuO were screened for catalytic activity; nano-CuO found most efficient. High to excellent amount of yields were reported. The catalyst reusability for five successive runs was also mentioned. The different active methylene compounds were used to afford polysubstituted quinolines.

Hosseini-Sarvari (2011) reported Friedlander synthesis of polysubstituted quinolines using nanoflake (NF-ZnO, Table 6) as a nanocatalyst under solvent free conditions [120]. The various metal oxides such TiO_2_, ZnO, MgO, CaO, commercial ZnO (CM-ZnO) and NF-ZnO were screened as catalyst for the reaction; among all CM-ZnO and NF-ZnO found efficient however NF-ZnO proved comparatively most efficient with short reaction times and higher yields. The article comprises rich library of quinoline scaffolds prepared from different active methylene compounds. The catalyst NF-ZnO was reused for two more subsequent cycles with almost consistent catalytic activity.

**Scheme 17:**
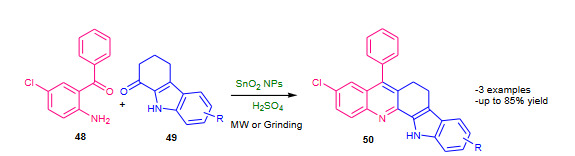
Nano-SnO2 catalysed modified Friedlander synthesis.

Roopan and Khan (2010) investigated synthesis of biologically active 9-chloro-6,13-dihydro-7-phenyl-5H-indolo [3,2-c]-acridine derivatives using SnO_2_ nanoparticles (nano-SnO_2, _Table 6) as a catalyst via a modified Friedlander synthesis under solvent free conditions [121]. The reaction mixture containing substituted amino ketone **48**, active methylene compound **49**, a drop of conc. H_2_SO_4_ and the catalyst SnO_2 _nanoparticles was microwave (500 W) irradiated or ground by mortar and pestle; affording good yields of corresponding acridine derivatives **50** (Scheme 17). The microwave protocol took shorter time but under grinding conditions yields were comparatively high. The article reported hemolytic activity study of synthesized polycyclic quinolines.

**Scheme 18:**
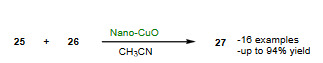
Nano-CuO catalyzed modified Friedlander synthesis.

Venkanna et al. (2014) performed synthesis of quinoline-2,3-dicarboxylates using nano-CuO powder (Table 6) as a catalyst in acetonitrile by modified Friedlander heteroannulation [122] (Scheme 18). Although the method seems conventional with use of solvent, longer reaction times but found to be reported high to excellent yields. The catalyst recovered was recycled for three consecutive cycles with gradual loss in activity after each cycle.

#### 3.5.4. Hybrid nanocatalysts

Ziyadi and Heydari (2014) attempted Friedlander quinolines synthesis using ferric nitrate supported polyvinylalcohol [PVA/Fe(NO_3_)_2, _Table 6] nanofiber mats as a heterogeneous catalyst [123]. High to excellent amount of yields were reported. The catalyst reusability for four more successive cycles was mentioned with almost retained catalytic activity. The article contributed a new form of hybrid catalyst for the improvement in the Friedlander reaction. The use of toxic solvent limits the credit of protocol and makes it align to conventional.

Godino-Ojer et al. (2017) demonstrated Friedlander synthesis using metal-doped carbon aerogels as a catalyst under solvent free conditions [124]. The different carbon aerogels were synthesized by polymerization of resorcinol (R) and formaldehyde (F) doping with transition metal nanoparticles. The nanocarbons doped with Co(0) and Cu(0) named RFCoS and RFCuS (Table 6), respectively. The catalyst systems were found to be efficient and affording high to excellent yields. The reusability of RFCuS catalyst was mentioned for two more subsequent cycles with consistent catalytic activity.

**Scheme 19:**
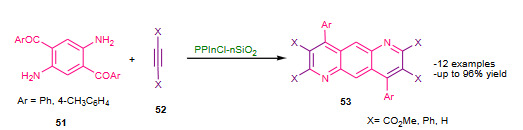
PPInCl-nSiO2 catalyzed modified Friedlander synthesis.

**Figure 11:**
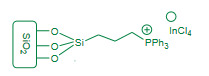
PPInCl-nSiO2 nanocatalyst.

Azizi et al. (2018) reported synthesis of quinolines and pyrido [3,2-g or 2,3-g]quinolines catalyzed propylphosphonium tetrachloroindate ionic liquid supported on nanosilica (PPInCl-nSiO_2_, Figure 11, Table 6) as a heterogeneous catalyst via a modified Friedlander heteroannulation under solvent free conditions [125]. The mixture of aminoketones **51**, alkynes **52**, and PPInCl-nSiO_2 _catalyst was heated solvent free conditions; affording high to excellent yields of substituted quinolines **53** (Scheme 19). The catalyst was reused for four consecutive cycles with consistent catalytic activity. The article shared new quinoline scaffolds and added PPInCl-nSiO_2_ as an efficient nanocatalyst to the Friedlander advancements. In spite of lengthy catalyst preparation, the protocol contributed to the literature.

#### 3.5.5.Miscellaneous nanocatalysts

Cho and Ren (2007) described nanopalladium (Nano-Pd, Table 6) catalysed modified Friedlander quinoline synthesis under conventional conditions [126]. The mixture of **29** and octyl aldehyde was heated in toluene in the presence of nano-Pd as a catalyst; affording 3-hexylquinoline along with considerable amount of oxazine. The catalyst used for two more successive cycles with noticeable decrement in the yield of 3-hexylquinoline and increment in yield of oxazine. The article added a new nanocatalyst but, the use of toxic solvent, poor yields, inefficient reusability of catalyst limits the credit of method. 

**Scheme 20:**
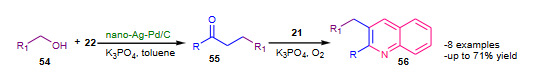
Nano-Ag-Pd/C catalyzed modified Friedlander synthesis.

Chen et al. (2013) achieved synthesis of quinolines through one pot, two step reaction using Ag-Pd alloy nanoparticles supported on carbon (Nano-Ag-Pd/C, Table 6) as a nanocatalyst [127]. The ketones **22** were coupled with primary alcohols **54** via hydrogen autotransfer process; yielded α-alkylated ketones **55** which further reacted with 2-aminobenzyl alcohols **21** via the modified Friedlander synthesis to afford polysubstituted quinolines **56** in moderate to good yields (Scheme 20). The reusability of catalyst was found inefficient due to considerable decrease in yield. The article proposed a modified Friedlander synthesis using nano-Ag-Pd/C catalyst though it involved use of toluene.

Angajala and Subashini (2015) reported solvent free Friedlander heteroannulation using nickel nanoparticles (NiNPs, Table 6) as a nanocatalyst [128]. The biosynthesis of nickel nano catalyst was achieved using literature method reported by the same group from *Aegle mearmelos *Correa aqueous leaf extract. The catalyst was reused for five more consecutive cycles with retained activity up to third cycle and thereafter it decreases considerably. The short reaction times, excellent yields, solvent free conditions, catalyst reusability highlights the advantages of the method over conventional Friedlander reaction.

Godino-Ojer et al. (2017) attempted solvent free Friedlander quinoline synthesis using carbon aerogels as a nanocatalyst under thermal as well as microwave conditions [129]. The two organic RF aerogels were prepared using resorcinol (R) and formaldehyde (F) with or without polymerization catalyst (Na_2_CO_3_) and activated by steam (S); labelled namely RFS, RFNa, and RFNaS, respectively. Also other carbon aerogels were prepared as RFS-PO and RFS-PS (RFS-PO indicates steam activated preparation without polymerization catalyst and oxidized with H_2_O_2_). The model Friedlander reaction was performed using all the prepared carbon aerogel-based catalyst systems such as RFS, RFS-PO, RFS-PS, RFNaS, RFNa-PO, RFNa-PS; among all RFS-PO, RFS-PS (Table 6) showed most efficient catalytic activity. The authors reported metal free synthesis of quinolines using carbon aerogels; microwave assisted methodology taken shorter time than thermal.

## 4. Conclusion and future perspective

The role of polymers in several** **forms, structure and nature within the** **development of Friedlander heteroannulation for the synthesis of novel quinoline scaffolds was reported in the literature. Around hundred articles are reviewed during this context which has involved the utilization of sort of** **polymers as a support to the reagent or catalyst support for the Friedlander reaction. Numerous attempts were noted, which are** **successfully contributed the remarkable modification also** **as advancement for the** **Friedlander reactions over the classical and traditional** **approaches. The few of research groups have successfully introduced synthesis of latest** **quinoline scaffolds via Friedlander cyclo-condensation with improved methodology. The massive** **number of protocols employed solvent free conditions, unconventional energy sources like** **a microwave irradiation, an ultrasonication; which have reported higher yields of desired poly-substituted quinolines. Polymers in hybrid forms as a catalyst were also reported with an efficient catalytic activity and reusability. Recently, in last decade; nanocatalysis has profoundly contributed the noticeable improvement over** **classical Friedlander reaction conditions with recyclability of catalyst especially with magnetic nanocatalyst and with simple workup** **procedures. Although, nanocatalysis proved itself as new and effective methodology; but, the preparation of nanocatalyst and its characterization seems costlier compare to another** **heterogeneous catalysts which can limit the precious** **impression of catalytic advancement. From the available literature, the advancements made up for** **Friedlander reaction which are remarkably noteworthy include simple heterogeneous workup** **protocol, reusability of polymeric catalyst, microwave assisted and solvent free conditions.

The researchers working in the** **field of polymer supported synthesis are challenged to develop new polymeric catalysts which may operate under moderate, low temperature** **reaction conditions with maximum productivity and recyclability. As per this** **literature, this might be the sole review which can** **highlight the role of polymers as a catalyst or as a catalyst or reagent support for the Friedlander heteroannulation; which can surely form an upscale and valuable resource for seekers in polymer supported synthesis of other biologically important heterocyclic scaffolds. Also, this review will** **help to encourage the researchers to develop greener synthetic approaches and may provide newer ideas to look and use new sorts of nanocatalysts. The broader application of suitable polymer supports can also be considered to achieve total synthesis of pharmacologically active natural heterocyclic motifs which have challenged upcoming researchers in the** **field.
